# Development and validation of a risk prediction model for painful diabetic peripheral neuropathy in type 2 diabetes mellitus: a multicenter retrospective study

**DOI:** 10.3389/fendo.2025.1651493

**Published:** 2025-11-27

**Authors:** Yanpi Li, Xiyun Wang, Huimin Hu, Xinyi Zhou, Naichong Hu, Wenhui Liu, Yi Zhang, Peng Mao, Liyuan Xu, Qian Zhu, Bifa Fan, Yifan Li

**Affiliations:** 1 Beijing University of Chinese Medicine, Beijing, China; 2 Department of Pain Management, China-Japan Friendship Hospital, Beijing, China; 3 Department of Pain Management, The Fourth Affiliated Hospital of Soochow University, Jiangsu, China

**Keywords:** machine learning, SHAP analysis, web-based nomogram, multivariable logistic regression, multicenter retrospective study

## Abstract

**Objective:**

To construct and validate a clinical model to predict painful diabetic peripheral neuropathy (PDPN) risk in type 2 diabetes mellitus (T2DM) patients for early identification and intervention in primary care.

**Methods:**

A total of 1,984 patients with T2DM were included in the analysis. After data preprocessing and application of the Synthetic Minority Oversampling Technique (SMOTE) with a 200% oversampling ratio, feature selection was performed using Least Absolute Shrinkage and Selection Operator (LASSO) regression with 10-fold cross-validation. Six predictive models: multivariable logistic regression (LR), random forest (RF), extreme gradient boosting (XGBoost), Light Gradient Boosting Machine (LightGBM), artificial neural network (ANN), and support vector machine (SVM)—were developed and tuned using repeated 5-fold cross-validation. Model performance was evaluated on the independent test cohort using comprehensive discrimination and calibration metrics. To enhance clinical interpretability, a nomogram and SHapley Additive exPlanations (SHAP) analysis were implemented to visualize predictor contributions.

**Results:**

Ten variables were selected as predictors. Among 1,984 patients, 81 (4.08%) had PDPN. LR model demonstrated the most favorable trade-off for screening purposes, with an area under the receiver operating characteristic curve (AUC-ROC) of 0.894 (95% CI: 0.814–0.964), area under the precision–recall curve (PR-AUC) of 0.470 (95% CI: 0.258–0.665), and balanced accuracy of 0.826 (95% CI: 0.667–0.932). SHAP analysis identified musculoskeletal disorders and HbA1c as the most influential predictors. A user-friendly dynamic web-based nomogram was constructed to support clinical implementation.

**Conclusion:**

We established and validated a clinically interpretable model for PDPN risk prediction in patients with T2DM. The dynamic nomogram enables individualized risk estimation and may assist timely intervention in routine practice.

## Introduction

Diabetic peripheral neuropathy (DPN) is one of the most prevalent chronic complication of type 2 diabetes mellitus (T2DM), affecting an estimated 30% to 90% of patients globally (1, 2). Its prevalence increases markedly with age and disease duration, posing a significant public health concern as populations age and diabetes incidence rises. Approximately 21.0% to 53.7% of DPN cases progress to painful DPN (PDPN) (3, 4), characterized by distal symmetric polyneuropathy and neuropathic pain (e.g. hyperalgesia, burning sensations, and electric-shock sensations) (3, 4). The prevalence of PDPN has been reported to range from 3.3% to 65.3%, influenced by variations in study methodology, diagnostic criteria, and patient population selection (5–7). Recent systematic reviews provide more precise estimates, with Tao et al. (8) reporting a pooled global prevalence of 46.7% (95% CI, 41.8–51.7) and Zhou et al. (9) estimating 33.9% (95% CI, 19.4–48.5).

The economic and quality-of-life burden of PDPN is considerable. Annual direct medical costs per patient have been estimated at USD 9,349 to 20,887, driven by hospitalization, long-term analgesic use, and management of related complications (10, 11). Additionally, PDPN is frequently associated with sleep disturbances (up to 70%) and depressive symptoms (30%~45%), resulting in a 40%~60% reduction in quality of life (12–14).

Current PDPN management remains suboptimal: fewer than one-third of patients achieve adequate pain relief with pharmacotherapy, largely due to the lack of pathophysiology-targeted agents (15). Moreover, PDPN is critically underdiagnosed (detection rate: ~50%), attributable to insidious symptom onset and the absence of standardized screening protocols in primary care (11, 16). Delayed diagnosis heightens risks of irreversible nerve damage, diabetic foot ulcers, and falls (13). Early identification of high-risk individuals is thus essential to enable timely intervention and potentially prevent the progression of disease.

Existing PDPN prediction models exhibit limited generalizability to Chinese populations owing to genetic, cultural, and healthcare system differences (15). Of the two published models, one was derived from Western cohorts, while the other (single-center Chinese study) lacks external validation and regional applicability (1, 17). No model has yet addressed the high-risk T2DM population in Beijing, where diabetes prevalence is rapidly rising.

To address this gap, we developed and validated a clinical prediction model for PDPN using multicenter data from Beijing. The model incorporates demographic and clinical variables, including glycemic control indicators and common comorbidities, to enable early risk stratification and support targeted screening in resource-limited settings.

## Methods

### Study design and participants

This was a multicenter retrospective cross-sectional study conducted between May 2023 and March 2024, involving 2,398 patients with T2DM from 13 community healthcare facilities in Beijing. Eligible participants were identified retrospectively from existing medical records, and subsequently invited to complete standardized questionnaires and undergo clinical assessments during a single study visit. The participating institutions included: Lisui Town Health Center, Chengqu Community Health Service Center, Tianzhu Town Health Center, Beixiaoying Community Health Service Center, Longshan Street Community Health Service Center, Wanshoulu Community Health Service Center, Financial Street Community Health Service Center, Ganjia Kou Community Health Service Center, Wanping Community Health Service Center, Wulidian Community Health Service Station, Yongxin Jiayuan Community Health Service Station, Honghuiyuan Community Health Service Station, Zhuanta Community Health Service Station. All cases reached a final definite diagnosis of either PDPN or Non-PDPN.

Inclusion criteria: (1) age ≥18 years; (2) diagnosed with T2DM.

Exclusion criteria: psychiatric or neurological disorders (e.g., cognitive impairment) potentially confounding PDPN assessment.

Patients diagnosed with PDPN met all the following criteria (18): (1) presence of distal symmetrical polyneuropathy, confirmed by a modified Toronto Clinical Neuropathy Score (mTCNS) of ≥5 (19); (2) presence of daily neuropathic pain for at least three months, verified by a Douleur Neuropathique 4 (DN4) questionnaire score of ≥4 (20).

The diagnosis of T2DM was established according to the World Health Organization (WHO) 1999 criteria, requiring either a fasting plasma glucose (FPG) ≥ 7.0 mmol/L, a 2-hour plasma glucose ≥ 11.1 mmol/L during a 75-g oral glucose tolerance test (OGTT), or a random plasma glucose ≥ 11.1 mmol/L in the presence of typical symptoms. For asymptomatic individuals, confirmation by repeated testing on a separate day was required.

After initial screening of 2,398 participants, 3 were excluded due to duplicate records, 164 due to unclear or non–T2DM diagnoses, and 247 due to missing data, leaving 1,984 eligible participants for the final analysis. The protocol was approved by the Ethics Committee of China-Japan Friendship Hospital (approval no. 2023-KY-343), with a waiver of informed consent due to the retrospective nature of the data analysis. Our research adheres to the ethical guidelines outlined in the Declaration of Helsinki (October 2013).

### Data collection and variables

A structured questionnaire was designed to collect information on potential risk factors associated with PDPN, covering demographic and lifestyle characteristics, glycemic control, and comorbid conditions. Demographic and lifestyle variables included age, sex, body mass index (BMI), blood pressure, occupation, education level (categorized as junior high school or below, high school/vocational/technical school, associate degree, bachelor’s degree, and postgraduate or above), marital status (unmarried, married, widowed, or divorced), smoking history (defined as smoking at least one cigarette per day for a cumulative duration of six months or more), alcohol consumption history (defined as consuming alcohol at least once per week for a continuous period of six months or longer), and physical activity level. Physical activity was classified into three categories based on occupational demands: low-intensity (≥75% of working time spent sitting or standing with minimal exertion, such as office workers, watch repairers, sales personnel, and hotel staff), moderate-intensity (≥75% of time engaged in physically active tasks, such as vehicle drivers and electricians), and high-intensity (≥60% of time spent performing strenuous physical labor, including manual agricultural workers, steelworkers, dancers, athletes, porters, and miners). Glycemic control variables included diabetes duration, most recent hemoglobin A1c (HbA1c), FPG (categorized as < 6 mmol/L, 6~7 mmol/L, 7~8 mmol/L, > 8 mmol/L), postprandial plasma glucose (PPG; < 8 mmol/L, 8~10 mmol/L, 10~11.1 mmol/L, > 11.1 mmol/L), adherence to blood glucose self-monitoring (defined as performing self-monitoring of blood glucose at least 80% of the frequency individually recommended by the treating physician), and antidiabetic treatment regimen (oral hypoglycemic agents, insulin injections, both, or none). For HbA1c, values measured within the 3 months prior to study enrollment were used. If multiple HbA1c measurements were available within this timeframe, the most recent value was selected. Comorbidities included metabolic syndrome, hypertension, cardiovascular disease, hyperlipidemia, rheumatology conditions, and musculoskeletal disorders.

### Model development, evaluation and interpretation

In this study, we employed stratified random sampling to divide the dataset into three distinct subsets: a training set (49% of the total sample), a validation set (21%), and an independent test set (30%). The training set was used in model development, the validation set for hyperparameter tuning and classification threshold optimization. The independent test set was reserved strictly for final model evaluation.

To address the class imbalance during model development, we applied the Synthetic Minority Oversampling Technique (SMOTE) exclusively to the minority class (PDPN+) in the training set, with a 200% oversampling ratio. All majority class (PDPN−) samples were retained to preserve clinical information. Feature selection was conducted on the balanced development dataset (training + validation) using the Least Absolute Shrinkage and Selection Operator (LASSO) regression with 10-fold cross-validation. Predictors with non-zero coefficients at the regularization parameter selected according to the one-standard-error (1-SE) rule were retained for subsequent modeling .

Based on the selected features, we developed a multivariable logistic regression (LR) model and five machine learning (ML) classifiers: Random Forest (RF), Extreme Gradient Boosting (XGBoost), Light Gradient Boosting Machine (LightGBM), Support Vector Machine (SVM) and Artificial Neural Network (ANN). To enhance model robustness and prevent overfitting, 5-fold cross-validation repeated 5 times was applied within the training and validation cohorts during model development and hyperparameter tuning. Hyperparameter optimization was performed using exhaustive grid search combined with repeated cross-validation within the training and validation cohorts. For each combination of hyperparameters, the area under the receiver operating characteristic curve (AUC-ROC) was used as the primary selection criterion. All final models were retrained using the optimal hyperparameters on the combined development data before independent evaluation. To ensure reliability, all model performance metrics are reported as the mean and 95% confidence interval (CI) derived from 1000 bootstrap resamplings of the test set.

Model performance was evaluated on the independent test set using a comprehensive set of metrics, including AUC-ROC, area under the precision–recall curve (PR-AUC), Brier score, G-mean, sensitivity, specificity, F1-score, recall, balanced accuracy, Matthews correlation coefficient (MCC) and decision curve analysis (DCA).

**Figure 1:**
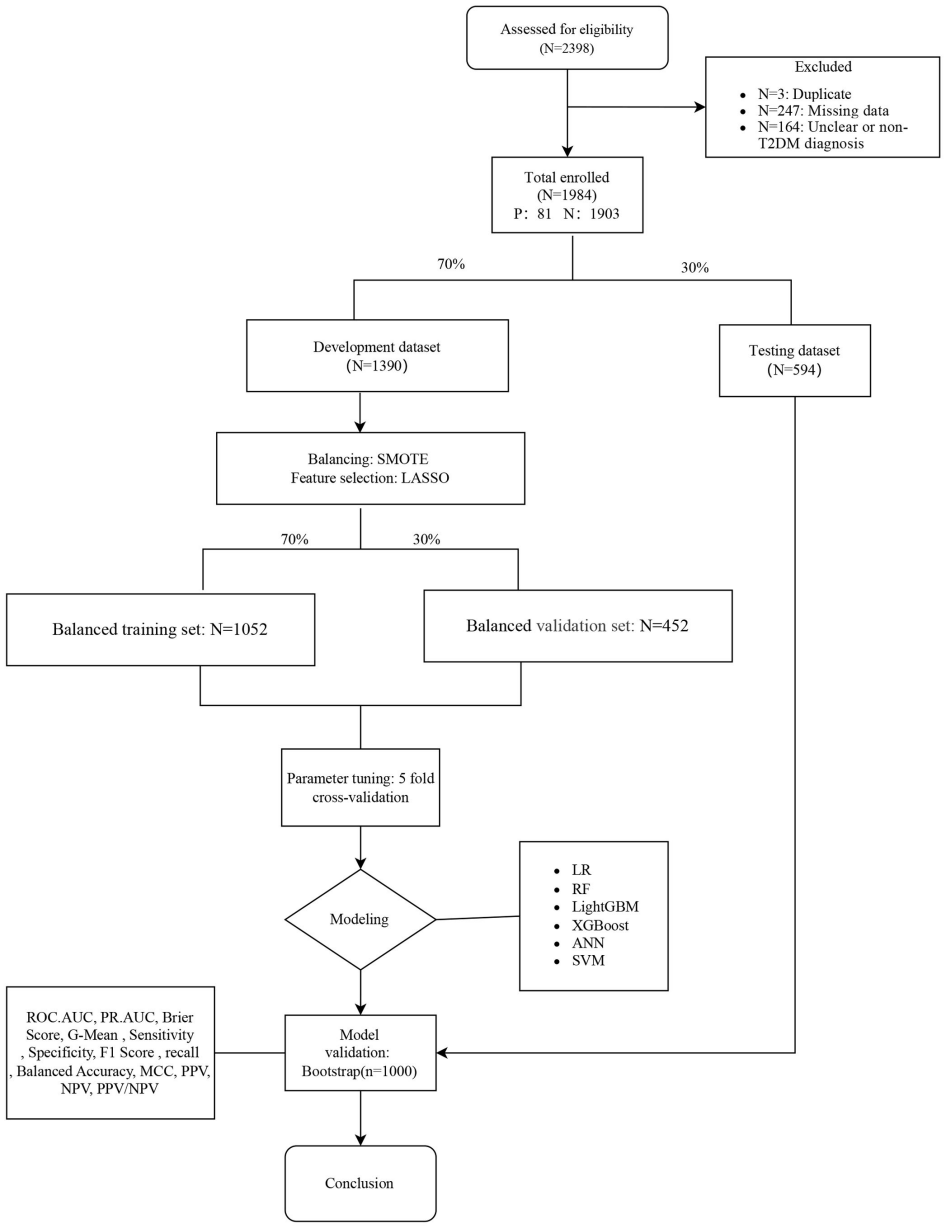
Workflow of data preprocessing, resampling, feature selection, model development, and validation.

To further strengthen the clinical applicability and interpretability of the proposed model, we constructed a nomogram derived from the LR analysis and deployed an interactive, web-based dynamic nomogram that delivers patient-specific risk estimates to support real-time clinical decision-making. In addition, we applied the SHapley Additive exPlanations (SHAP) framework to compute and visualize feature-attribution plots for each model, thereby providing transparent, model-agnostic insights into variable importance. The complete modeling workflow is depicted in **Figure 1**.

### Statistical analysis

Patients were divided into PDPN and non-PDPN groups according to PDPN status. Between-group differences were analyzed using chi-square, t-tests, or Mann-Whitney U tests. Continuous variables were described as mean ± standard deviation for normally distributed data, or as median and interquartile range (Q1, Q3) for non-normally distributed data. Categorical variables were reported as counts (n) and corresponding percentages (%).

All analyses were conducted using R (version 4.5.0) and Python (version 3.13). Statistical significance was defined as a two-sided p-value less than 0.05.

## Results

### Baseline characteristics of study population

A total of 1,984 patients with T2DM were included in the final analysis, comprising 959 males (48%) and 1,025 females (52%). Of these, 1,903 patients (95.92%) did not develop PDPN, while 81 patients (4.08%) were diagnosed with PDPN. The patients were randomly divided into a development set (n = 1,390; 70%) and an independent test set (n = 594; 30%). Baseline characteristics were compared between the two sets. Except for rheumatology conditions, which showed a statistically significant difference, all other characteristics were comparable between the groups. In the development set, 57 patients (4.10%) developed PDPN, whereas 24 patients (4.04%) in the test set developed PDPN. **Table 1** summarizes the key baseline characteristics of the participants, while the detailed characteristics are provided in **Supplementary Table 1**.

**Table 1:** Baseline characteristics of participants with T2DM.

Variables	Total (n = 1984)	Test set (n = 594)	Development set (n = 1390)	Statistic	*P*
Age, M (Q_1_, Q_3_)	64.00 (57.00, 70.00)	64.00 (57.00, 70.00)	65.00 (57.00, 70.00)	Z=-0.53	0.593
Sex, n(%)				χ²=1.92	0.166
male	959 (48.34)	273 (45.96)	686 (49.35)		
female	1025 (51.66)	321 (54.04)	704 (50.65)		
BMI, M (Q_1_, Q_3_)	25.39 (23.44, 27.68)	25.39 (23.34, 27.72)	25.39 (23.44, 27.68)	Z=-0.04	0.967
Duration, M (Q_1_, Q_3_)	98.00 (45.00, 163.00)	93.00 (40.25, 158.00)	98.00 (48.00, 164.00)	Z=-1.78	0.075
HbA1c, M (Q_1_, Q_3_)	6.50 (6.00, 7.00)	6.50 (6.00, 7.00)	6.50 (6.00, 7.00)	Z=-1.02	0.309
FPG, n(%)				χ²=6.31	0.098
<6 mmol/L	268 (13.51)	87 (14.65)	181 (13.02)		
6–7 mmol/L,	942 (47.48)	288 (48.48)	654 (47.05)		
7–8 mmol/L	521 (26.26)	135 (22.73)	386 (27.77)		
>8 mmol/L	253 (12.75)	84 (14.14)	169 (12.16)		
PPG, n(%)				χ²=1.24	0.744
<8 mmol/L	433 (21.82)	139 (23.40)	294 (21.15)		
8–10 mmol/L	905 (45.61)	265 (44.61)	640 (46.04)		
10–11.1 mmol/L	397 (20.01)	117 (19.70)	280 (20.14)		
>11.1 mmol/L	249 (12.55)	73 (12.29)	176 (12.66)		
Antidiabetic treatment regimen, n(%)				χ²=0.91	0.824
oral hypoglycemic agents	1491 (75.15)	452 (76.09)	1039 (74.75)		
insulin	91 (4.59)	29 (4.88)	62 (4.46)		
both	259 (13.05)	73 (12.29)	186 (13.38)		
none	143 (7.21)	40 (6.73)	103 (7.41)		
PDPN, n(%)				χ²=0.00	0.950
yes	1903 (95.92)	570 (95.96)	1333 (95.90)		
no	81 (4.08)	24 (4.04)	57 (4.10)		

Z, Mann-Whitney test; χ², Chi-square test; M, Median; Q_1_, 1st Quartile; Q_3_, 3rd Quartile; BMI, Body Mass Index; HbA1c, Hemoglobin A1c; FPG, Fasting Plasma Glucose; PPG, Postprandial Plasma Glucose; SBP, Systolic Blood Pressure; DBP, Diastolic Blood Pressure; PDPN, Painful Diabetic Peripheral Neuropathy.

### Feature selection and model development

**Figure 2:**
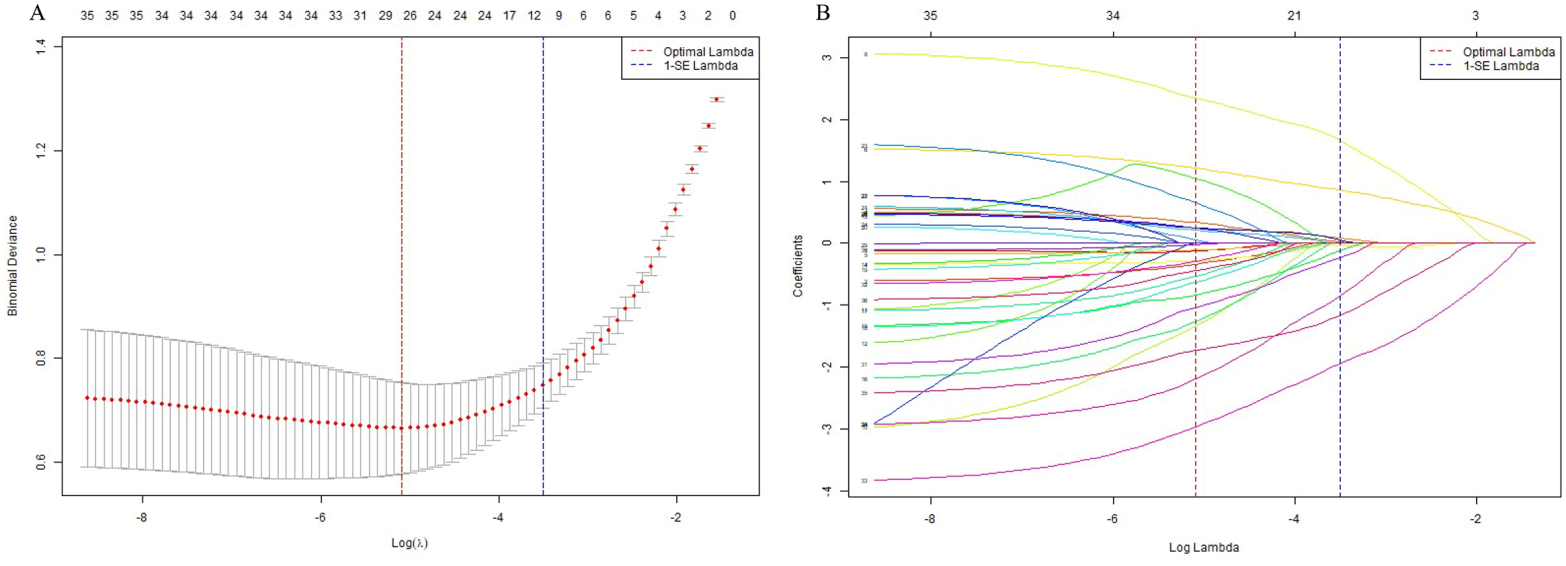
Feature selection using LASSO regression. **(A)** Plot of cross-validation error versus log(λ). The vertical dashed lines indicate the optimal values of the regularization parameter λ: λ.min (red), which gives the minimum mean cross-validated error, and λ.1se (blue), the most regularized model within one standard error of the minimum. **(B)** LASSO coefficient profiles of the selected features. Each curve represents the trajectory of a feature’s coefficient as the regularization parameter λ changes.

LASSO regression was performed in the balanced development set to automatically select important features (see **Figure 2**). By adjusting the regularization coefficient lambda (λ), LASSO regression reduces the loss function (binomial deviation) and shrinks the coefficients of less predictive variables to zero. Ten of the available features were identified as the most predictive at a shrinkage parameter (lambda.1se) of 0.03007005. The selected features were musculoskeletal conditions (β = 1.95), antidiabetic treatment regimen involving both oral hypoglycemic agents and insulin injections (β = 1.66), antidiabetic treatment regimen with oral hypoglycemic agents alone (β = −0.03), hyperlipidemia (β = 1.17), HbA1c (β = 0.86), neurological conditions (β = 0.85), hypertension (β = 0.24), FPG levels within the range of 7–8 mmol/L (β = −0.12), BMI (β = 0.09), sex (β = 0.05), and diastolic blood pressure (DBP; β = 0.02). Among these predictors, musculoskeletal conditions, combined antidiabetic therapy, hyperlipidemia, and elevated HbA1c levels emerged as the most significant positive predictors of PDPN, whereas moderate FPG levels showed a weak negative association. These variables were subsequently included in the machine learning model. For the LR model, 81 PDPN events and 10 retained predictors yielded an event-per-variable (EPV) ratio of 8.1.

### Development and comparison of prediction models

**Figure 3:**
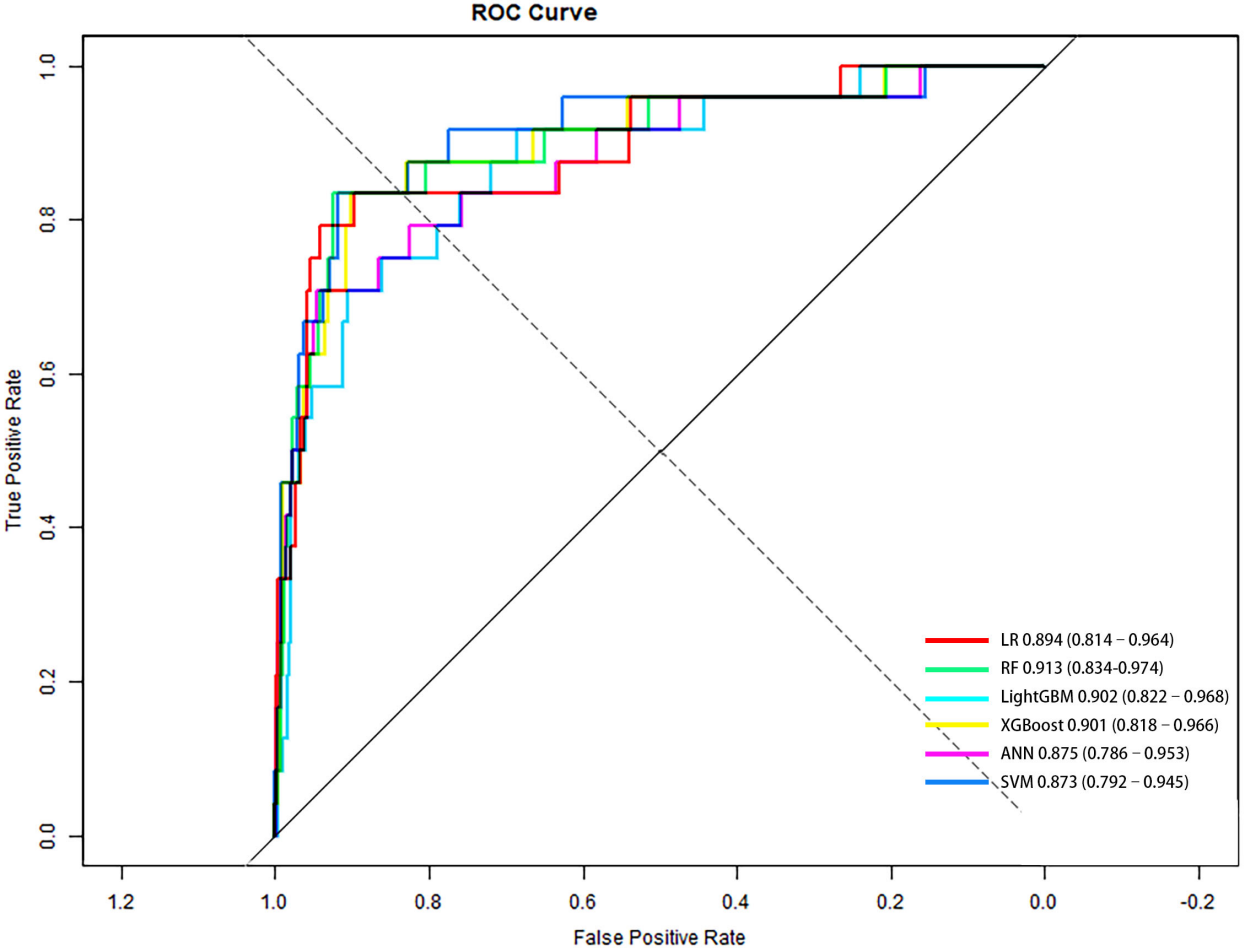
ROC curves of six predictive models for PDPN in the independent test set (n = 594). Diagonal line represents no discrimination (AUC = 0.5). Higher AUC indicates better performance; RF achieved the highest AUC. AUC and 95% CI for each model were: LR, 0.894 (0.814–0.964); RF, 0.913 (0.834–0.974); LightGBM, 0.902 (0.822–0.968); XGBoost, 0.901 (0.818–0.966); ANN, 0.875 (0.786–0.953); SVM, 0.873 (0.792–0.945).

Based on clinically and statistically significant features selected via LASSO regression, six predictive models were developed and evaluated: LR, RF, LightGBM, XGBoost, ANN, and SVM. Except for LR, hyperparameter tuning was performed via grid search with five-fold cross-validation on the training set to maximize mean AUC-ROC. The optimal parameters are summarized in **Table 2**. Model performance was comprehensively evaluated on the independent test cohort using a bootstrap resampling procedure (B = 1000), with all results reported as means and 95% CI. A summary of all model evaluation metrics is provided in **Table 3**. **Figure 3** shows the ROC curves for the 6 models, while **Supplementary Figure 1** presents DCA to better capture model performance in the context of low prevalence.

**Table 2:** Optimal parameters for five ML models in predicting PDPN.

Models	Optimal parameter
LR	–
RF	n_estimators=1000, max_features=2, min_samples_leaf=3, criterion="gini", bootstrap=True, random_state=42
LightGBM	objective binary, num_leaves 32, learning_rate 0.03, lambda_l2 5, num_threads 15, nrounds 145
XGBoost	nrounds = 300, max_depth = 5, eta = 0.05, gamma = 0.00, colsample_bytree = 0.60, min_child_weight = 1, subsample = 0.80, scale_pos_weight = 24.6, alpha = 0.01, objective = "binary:logistic"
ANN	size 5, decay 0.1, maxit 500, maxit 500^*^
SVM	C=10.000, sigma=0.100

“size = 5” indicates the number of neurons in the hidden layer, “decay = 0.1” represents the weight decay parameter (L2 regularization), and “maxit = 500” refers to the maximum number of training iterations.

LR, multivariable Logistic Regression; RF, Random Forest; LightGBM, Light Gradient Boosting Machine; XGBoost, Extreme Gradient Boosting; ANN, Artificial Neural Network, SVM, Support Vector Machine

**Table 3:** Predictive performance of six models in identifying PDPN.

Models	ROC.AUC(95% CI)	PR.AUC(95% CI)	Brier Score	G-Mean	Sensitivity	Specificity	PPV (Precision)	NPV	PPV/NPV	F1 Score	recall	Balanced Accuracy	MCC
LR	0.894 (0.814–0.964)	0.470 (0.258–0.665)	0.038 (0.027–0.051)	0.807 (0.577–0.932)	0.688 (0.333–0.913)	0.965 (0.936–0.998)	0.514 (0.306-0.923)	0.987 (0.973-0.996)	0.521	0.549 (0.414–0.686)	0.695	0.826 (0.667–0.932)	0.541
RF	0.913 (0.834-0.974)	0.488 (0.276-0.694)	0.029 (0.021-0.039)	0.744 (0.591-0.889)	0.571 (0.353-0.833)	0.983 (0.936-0.998)	0.652 (0.333-0.941)	0.982 (0.970-0.991)	0.664	0.582 (0.419-0.739)	0.575	0.777 (0.672-0.892)	0.56
LightGBM	0.902 (0.822–0.968)	0.413 (0.218–0.615)	0.031 (0.022–0.042)	0.772 (0.634–0.907)	0.620 (0.409–0.879)	0.971 (0.923–0.991)	0.504 (0.300-0.714)	0.984 (0.973-0.995)	0.512	0.542 (0.389–0.684)	0.624	0.796 (0.697–0.907)	0.525
XGBoost	0.901 (0.818–0.966)	0.421 (0.224–0.622)	0.031 (0.019–0.045)	0.680 (0.525–0.824)	0.475 (0.278–0.688)	0.988 (0.966–0.997)	0.639 (0.370-0.875)	0.978 (0.966-0.990)	0.653	0.535 (0.353–0.714)	0.475	0.731 (0.633–0.838)	0.529
ANN	0.875 (0.786–0.953)	0.427 (0.210–0.635)	0.036 (0.025–0.048)	0.745 (0.574–0.888)	0.579 (0.333–0.826)	0.973 (0.936-0.998)	0.527 (0.296-0.875)	0.982 (0.971-0.993)	0.537	0.526 (0.379–0.667)	0.585	0.776 (0.661–0.891)	0.509
SVM	0.873 (0.792–0.945)	0.290 (0.151–0.467)	0.037 (0.025–0.050)	0.710 (0.558–0.838)	0.524 (0.318–0.737)	0.972 (0.944–0.989)	0.460 (0.259-0.667)	0.980 (0.968-0.990)	0.469	0.481 (0.316–0.630)	0.525	0.748 (0.649–0.848)	0.349

LR, multivariable Logistic Regression; RF, Random Forest; LightGBM, Light Gradient Boosting Machine; XGBoost, Extreme Gradient Boosting; ANN, Artificial Neural Network; SVM, Support Vector Machine; AUC-ROC, Area Under the Receiver Operating Characteristic Curve; CI, Confidence Interval; PR-AUC, Area Under the Precision-Recall Curve; PPV, Positive Predictive Value; NPV, Negative Predictive Value; MCC, Matthews Correlation Coefficient.

Among all models, LR demonstrated the most favorable profile for screening purposes. Although its PR-AUC (0.470, 95% CI: 0.258–0.665) and AUC-ROC (0.894, 95% CI: 0.814–0.964) were slightly lower than those of more complex models, LR achieved the highest sensitivity (0.688), G-Mean (0.807), and balanced accuracy (0.826). These metrics indicate a strong ability to detect true positive cases while maintaining reasonable specificity (0.965). Importantly, LR also yielded a PPV of 0.514 and an NPV of 0.987, highlighting its clinical utility as a screening tool to effectively rule out PDPN. Additionally, the interpretability and simplicity of LR further support its utility in clinical settings where early identification is prioritized, particularly given the constraints of a limited number of events. DCA confirmed that the LR model provided greater net benefit than both the “treat-all” and “treat-none” strategies across a low-risk threshold range (0.01–0.30), reinforcing its value for early risk screening where sensitivity is paramount.

The RF model yielded the highest overall discrimination with a PR-AUC of 0.488 and AUC-ROC of 0.913, alongside the lowest Brier score (0.029), suggesting well-calibrated risk probabilities. RF performed particularly well on PPV (0.652) and NPV (0.982), reflecting reliable case identification in positive predictions. However, sensitivity was lower (0.571), making RF less optimal for screening contexts where minimizing missed cases is critical. **Supplementary Figure 2** presents the DCA, which demonstrated that the RF model consistently yielded greater net benefit across a clinically relevant threshold range (0.01–0.25), highlighting its potential value for risk stratification and for reducing unnecessary interventions.

LightGBM showed balanced performance, with PR-AUC 0.413 and ROC-AUC 0.902. PPV and NPV were 0.504 and 0.984, respectively, demonstrating reliable negative prediction but only moderate positive predictive value. Sensitivity (0.620) was relatively strong, suggesting LightGBM may be useful where case detection is prioritized.

XGBoost achieved a ROC-AUC of 0.901 and a PR-AUC of 0.421. Its PPV was relatively high (0.639), alongside an NPV of 0.978, and it achieved the highest specificity (0.988). However, recall was the lowest (0.475), limiting its utility as a first-line screening tool despite strong calibration and high precision.

ANN achieved a ROC-AUC of 0.875 and PR-AUC of 0.427. The PPV was 0.527 and NPV 0.982, indicating acceptable classification balance. Sensitivity was 0.579 and specificity 0.973, suggesting ANN provided a moderate trade-off but did not exceed LR in clinically critical measures.

The SVM model performed the worst overall, with the lowest PR-AUC (0.290), F1 score (0.481), and MCC (0.349), limiting its suitability for clinical use in this context.

Taken together, LR provides the most clinically appropriate balance of sensitivity and overall classification performance for early PDPN risk screening, particularly in settings where underdiagnosis remains a concern. It should also be noted that the relatively small number of PDPN events may have limited the statistical power of more complex models such as ANN, RF, and XGBoost, potentially underestimating their true performance. As a sensitivity analysis, we repeated the random split of training and test sets. Results remained consistent (see **Supplementary Table 2**), supporting the stability of our findings.

### Model interpretation

**Figure 4:**
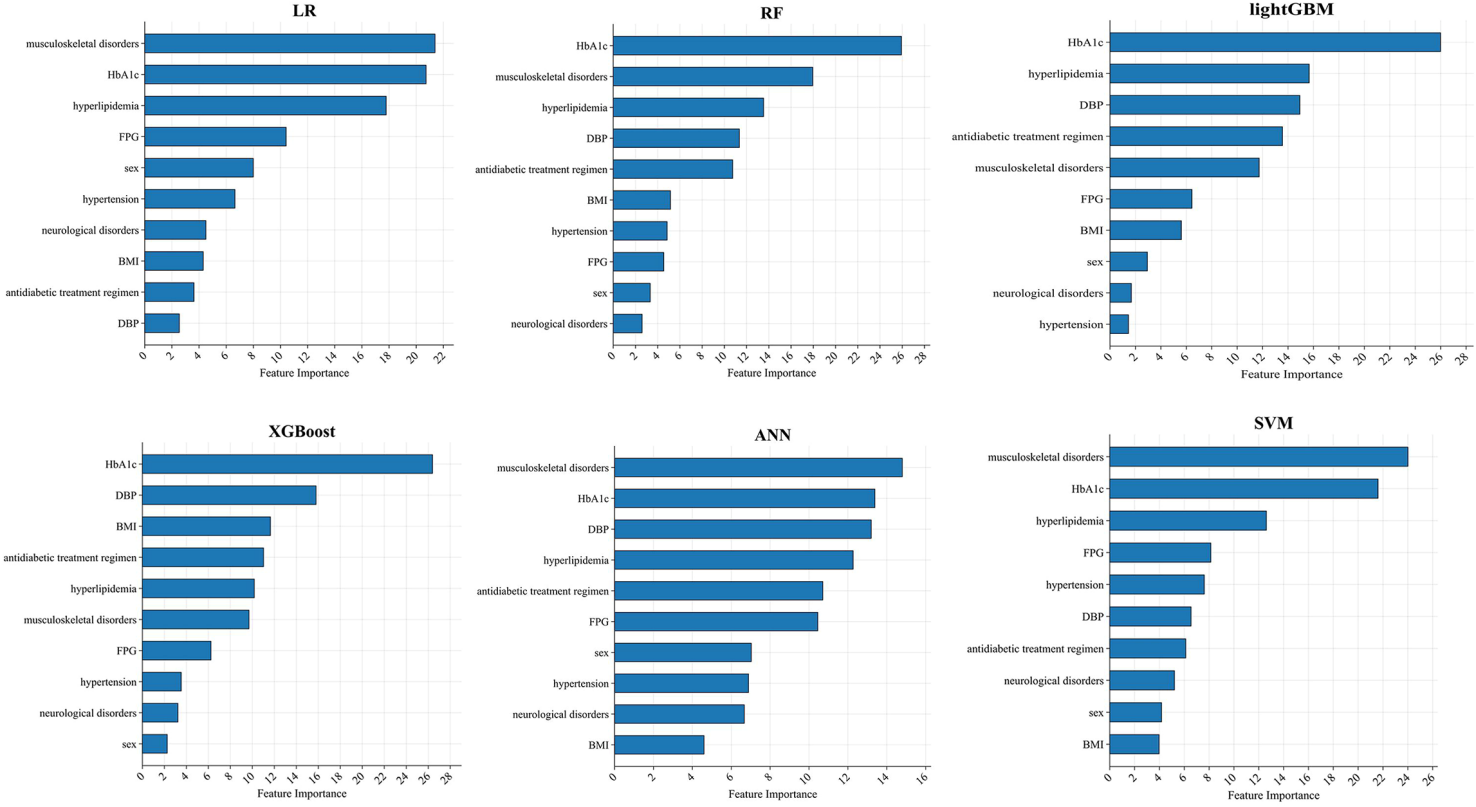
SHAP importance score ranking of the top 10 predictive features in the independent test set (n = 594). Key predictors include HbA1c, musculoskeletal disorders, DBP, hyperlipidemia, and BMI.

**Figure 5:**
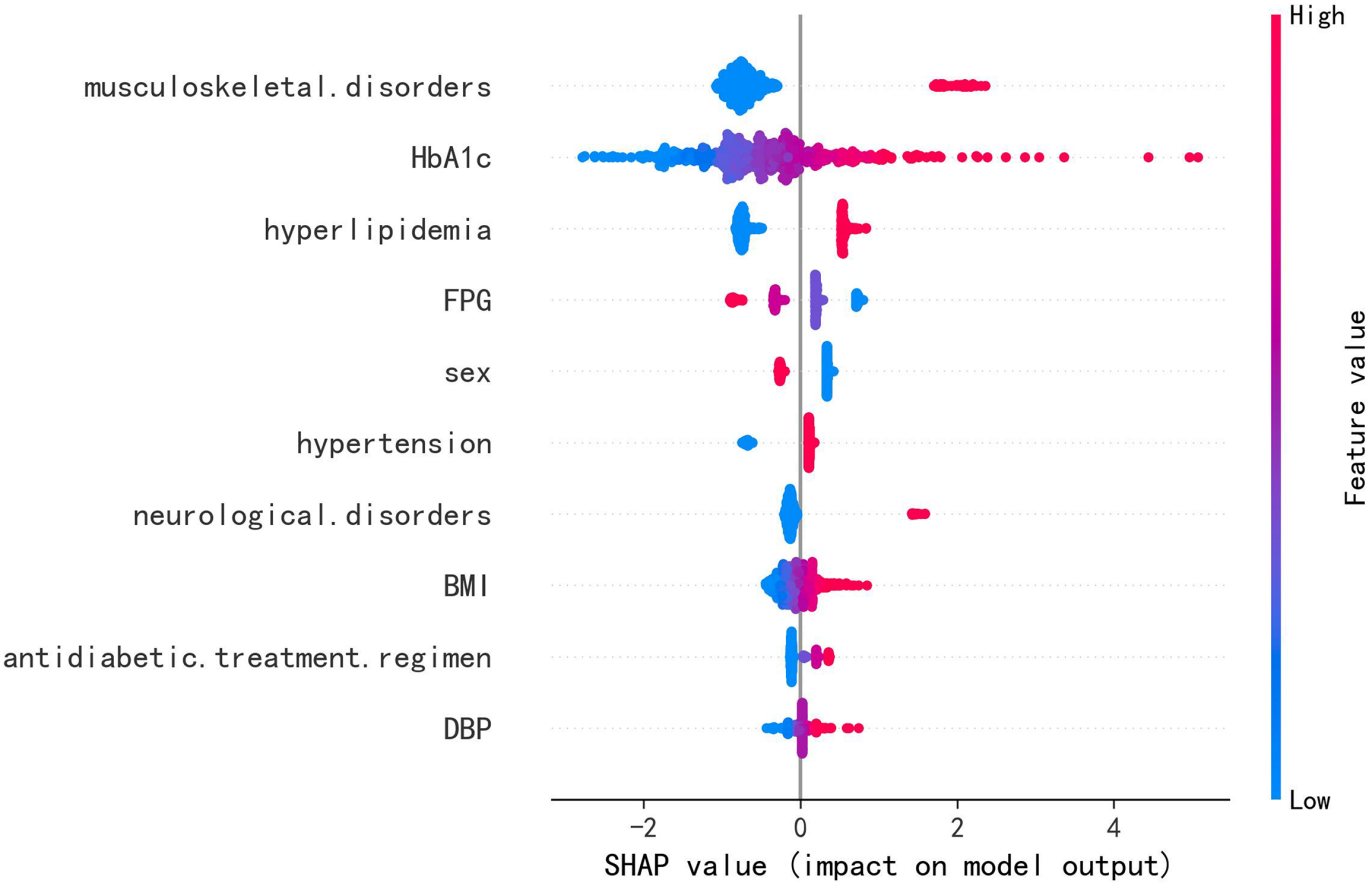
SHAP summary plot of the LR model in the independent test set (n = 594). Each dot represents an individual patient, with color indicating the feature value: red for higher or positive values, and blue for lower or negative values. The horizontal spread of points reflects the magnitude of each feature’s contribution to the model output; greater dispersion indicates a stronger impact.

Across all six algorithms, musculoskeletal disorders and HbA1c were the strongest predictors of PDPN, followed—without a consistent order—by DBP, hyperlipidemia, and BMI (see **Figure 4**). The direction and magnitude of each predictor’s effect in the logistic regression model were further evaluated using a SHAP summary plot with overlaid violin distributions (see **Figure 5**). Predictors are ranked vertically by mean absolute SHAP value, representing their global importance in the model. Each point denotes a single patient’s contribution, with color reflecting the feature value (blue: low, red: high). SHAP values > 0 indicate a positive impact on predicted PDPN risk, while values < 0 suggest a protective effect. The width of each violin plot at a given SHAP value corresponds to the density of data points with that level of impact. Musculoskeletal disorders demonstrated the widest SHAP range (approximately –3 to +5). This suggests that their presence typically increases PDPN risk, but in some contexts, the effect may be neutral or even protective. HbA1c, BMI, and DBP showed consistently positive SHAP values, suggesting that higher levels are associated with increased PDPN risk. Hyperlipidemia, female sex, hypertension, and neurological disorders also contributed positively, though with narrower SHAP distributions. FPG exhibited a negative association with PDPN risk, where higher values corresponded to lower SHAP scores. Finally, the antidiabetic treatment regimen revealed that patients receiving insulin had the highest model-estimated risk for PDPN.

**Figure 6:**
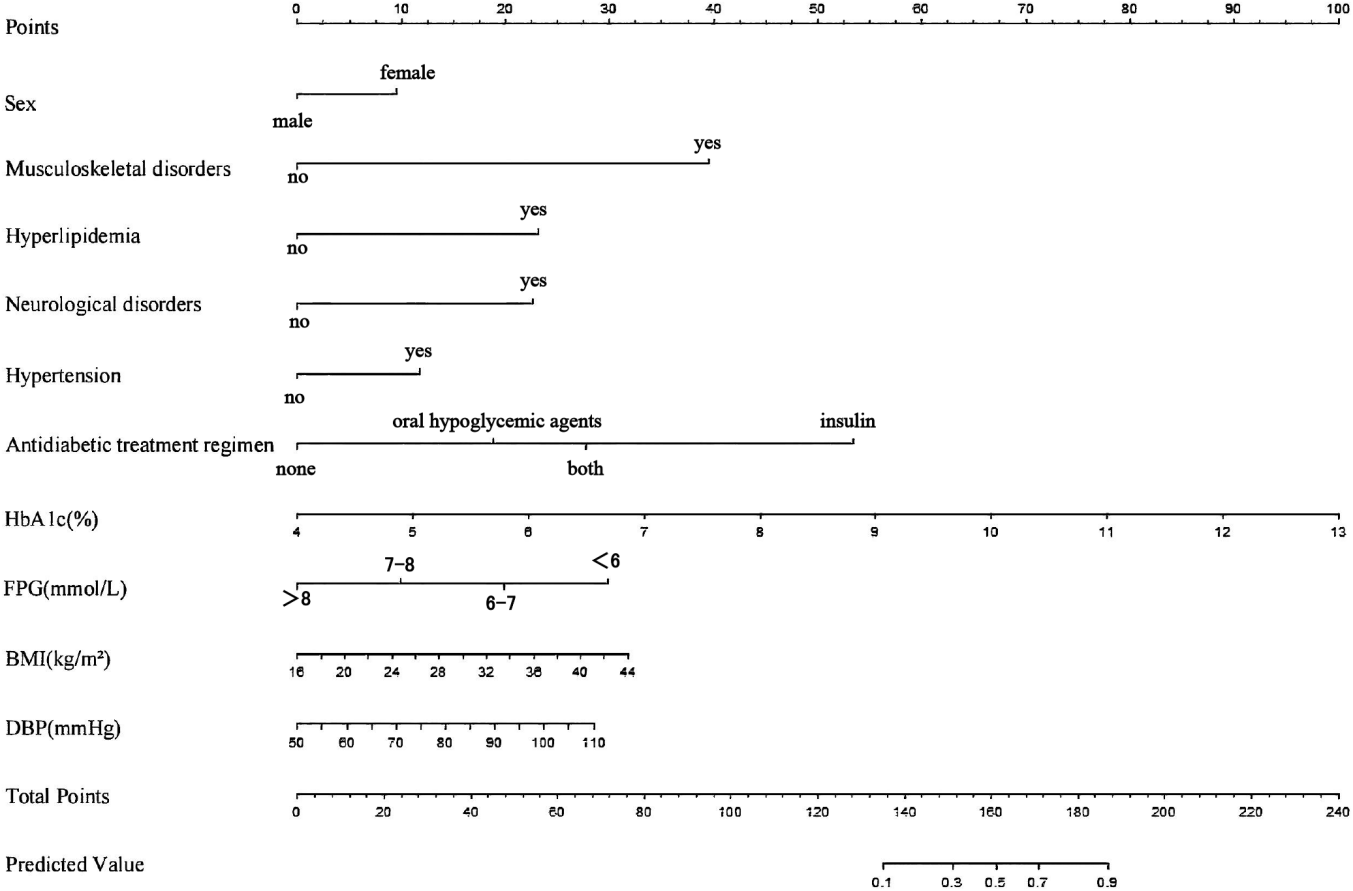
Nomogram of the LR model. For each variable, a vertical line is drawn upward to the “Points” row to determine its assigned score. The total score, obtained by summing all variable points, is then projected downward to the “Total Points” row to estimate the predicted probability of PDPN.

**Figure 7:**
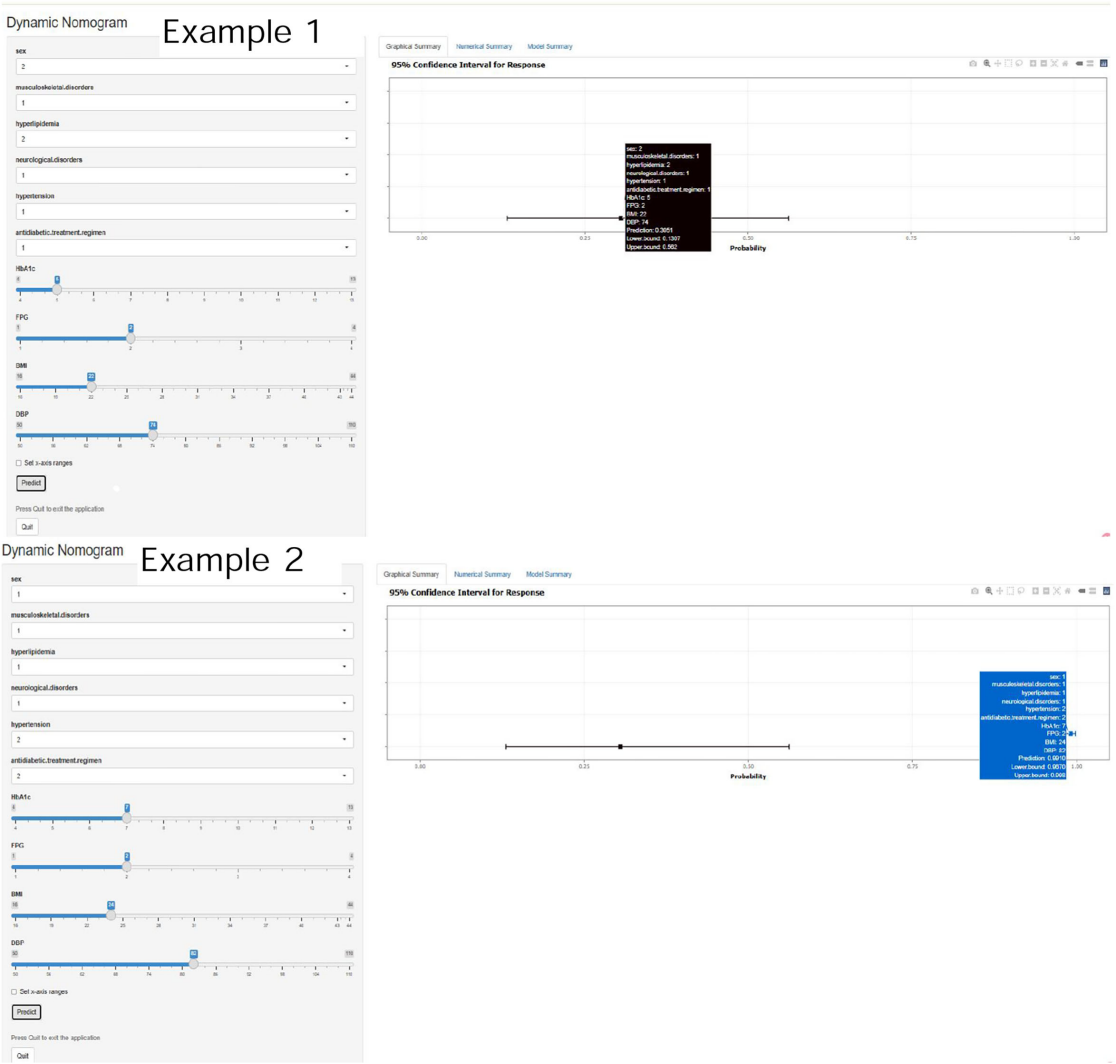
Representative screenshots of the web-based dynamic nomogram. Showing input controls for categorical and continuous predictors on the left. Individualized predicted probability with 95% confidence interval on the right. Tooltip displays current inputs, prediction results, and CI bounds.

The logistic-regression nomogram (see **Figure 6**) assigns point values to each of the ten variables; the sum of these points is converted directly to an individual probability of PDPN. Thus, clinicians can estimate risk simply by adding the scores for a patient’s characteristics. For bedside application, we have created an interactive web-based version of the nomogram https://ganzhi.shinyapps.io/PDPN/; temporary access is available from the corresponding author. **Figure 7** shows representative screenshots. In the first example, a female patient with musculoskeletal and neurological disorders, hypertension, no hyperlipidemia, oral antidiabetic therapy, an HbA1c of 5%, FPG of 6~7 mmol/L, BMI of 22 kg/m², and DBP of 74 mmHg has an estimated risk of 31%. In contrast, a male patient with musculoskeletal disorders, hyperlipidemia, and neurological disorders, no hypertension, insulin-only therapy, an HbA1c of 7%, FPG of 6–7 mmol/L, BMI of 24 kg/m², and DBP of 82 mmHg has an estimated PDPN risk of 99%. These examples underscore the nomogram’s ability to translate routinely collected clinical information into intuitive, patient-specific risk estimates that can be accessed on any smartphone or computer.

## Discussion

In this study, we developed and validated six clinical prediction models for PDPN among patients with T2DM, using multicenter community-based data from Beijing. The prevalence of PDPN in our cohort was 4.08%, markedly lower than reported in many tertiary care studies (1). This discrepancy may reflect differences in study settings, as our data were collected from primary care populations where patients typically present with milder or earlier-stage disease. Variability in diagnostic criteria, screening intensity, and the likelihood of under-recognition in primary care settings may also contribute. These findings underscore the importance of enhanced PDPN screening strategies in community-based populations. In addition, by leveraging multicenter community-based data, our work extends beyond existing single-center or tertiary-hospital models and reflects the real-world risk profile of Chinese primary care populations.

Ten variables were selected via LASSO regression for model development: musculoskeletal disorders, antidiabetic treatment regimen, hyperlipidemia, HbA1c, neurological conditions, hypertension, FPG, BMI, sex, and DBP. The resulting EPV of 8.1 is slightly below the traditional rule-of-thumb of 10, but still within an acceptable range supported by prior methodological studies (21, 22). However, overfitting risk was robustly mitigated through LASSO feature selection, bootstrap resampling, and independent test set validation, supporting the stability of our findings.

Notably, this study is among the first to incorporate antidiabetic treatment regimen as a predictive factor for PDPN. The inclusion of musculoskeletal and rheumatologic disorders, together with common comorbidities, further distinguishes this model and enhances its potential clinical applicability in early risk stratification.

Of the six models developed, LR and RF demonstrated superior performance. While RF yielded the highest AUC-ROC and PR-AUC, LR showed the highest sensitivity (0.688), G-Mean (0.807), and balanced accuracy (0.826), making it more suitable for pre-screening applications. Moreover, LR’s interpretability and ease of clinical integration, particularly through its transformation into a web-based nomogram, further support its practical utility in frontline healthcare settings.

The pathophysiology of PDPN remains incompletely understood and is likely multifactorial, involving metabolic, vascular, inflammatory, and neural mechanisms (18, 23). Genetic, psychological, and sociocultural factors may also contribute to disease susceptibility and symptom expression (12). We observed that patients with PDPN had a greater burden of comorbid conditions than those without. However, due to the cross-sectional nature of our study, we cannot determine causal directionality.

Musculoskeletal disorders emerged as a key predictor of PDPN in our model, with SHAP values ranging from strongly positive to mildly negative, suggesting a heterogeneous and context-dependent relationship. The risk-enhancing effects are likely mediated by inflammatory and mechanical pathways. Chronic conditions such as osteoarthritis and cervical spondylosis elevate systemic IL-6 and TNF-α levels (24, 25), both of which are implicated in neuropathic pain pathogenesis (26). Structural abnormalities like joint instability and spinal degeneration may also aggravate nerve damage via mechanical compression, compounding metabolic or ischemic injury (27). Epidemiological data show that nearly half of T2DM patients have coexisting arthritis, supporting this link. Conversely, DPN may contribute to musculoskeletal decline through muscle atrophy, impaired contractility, altered gait, and biomechanical imbalances—further accelerating bone loss and joint degeneration (28, 29). This bidirectional relationship may reflect a feedback loop between neuropathic and musculoskeletal pathology.

In contrast, the negative SHAP values observed in some individuals may reflect earlier healthcare engagement. Patients with musculoskeletal complaints are more likely to access care, potentially leading to earlier recognition and management of diabetic complications. Moreover, standard therapies—such as physical rehabilitation, anti-inflammatory agents, and vitamin D supplementation—may confer incidental neuroprotective effects (30). Overall, the observed association likely reflects overlapping pathophysiological domains between PDPN and musculoskeletal disorders, including neuroinflammation, metabolic stress, and impaired neuromuscular control. Further prospective studies are needed to clarify causality and underlying mechanisms.

Elevated HbA1c was another strong predictor of PDPN. Chronic hyperglycemia promotes neuronal damage and pain hypersensitivity via multiple molecular pathways, including MAPK and PKC signaling and systemic inflammation (31–33). Numerous studies have confirmed the association between HbA1c and both painful and painless DPN subtypes (34, 35).

Hyperlipidemia also contributed positively to PDPN risk in our model. This observation is consistent with established pathophysiological mechanisms, whereby elevated levels of free fatty acids in hyperlipidemic states undergo β-oxidation, leading to peripheral nerve injury—particularly affecting Schwann cells (36–39). The resulting oxidative stress, driven by reactive oxygen species (ROS), together with macrophage-mediated systemic and local inflammation, promotes the production of pro-inflammatory cytokines and chemokines. These processes, in turn, exacerbate neural damage (40, 41).

Unexpectedly, higher FPG levels were associated with a lower risk of PDPN in our dataset, contrary to most previous reports. Several explanations may account for this paradox. First, recall bias cannot be excluded, as FPG was self-reported. Second, lower current FPG may not reflect better long-term glycemic control but greater glycemic variability. Patients with lower fasting values can still experience substantial postprandial excursions, which have been shown to induce greater oxidative stress and neuronal injury than sustained hyperglycemia (42–44). Third, PDPN involves multiple overlapping pathogenic pathways (e.g., oxidative stress, neuroinflammation) driven by chronic hyperglycemia and glycemic excursions — factors not captured by a single FPG measurement (45). Fourth, intensive glucose-lowering therapy may paradoxically induce neuropathic pain in some patients (46). Finally, reverse causation and confounding are possible, as patients with PDPN may receive more intensive treatment or adhere more strictly to regimens, resulting in lower FPG despite prolonged dysglycemia.

Hypertension has been identified in multiple studies as an independent risk factor for DPN (47). Experimental models further support this association. Studies comparing hypertensive diabetic rats with normotensive diabetic controls have demonstrated more severe neuropathic damage, including reduced nerve perfusion, heightened oxidative stress, decreased Schwann cell density, axonal atrophy, and small fiber degeneration (47, 48).

BMI exhibited a nonlinear yet notable impact, with higher values (represented by red points) clustering on the positive end of the SHAP axis. This indicates that increased BMI is associated with elevated PDPN risk—a finding consistent with population-based studies across multiple countries, where obesity is commonly observed in DPN cohorts (49, 50). In contrast, sex (female) demonstrated a modest clustering near zero SHAP values, indicating a limited direct contribution within the model. Nevertheless, this pattern is consistent with clinical observations: women are less likely to exhibit objective signs of neuropathy but more frequently report painful DPN symptoms compared to men (15). This apparent paradox may reflect sex-specific differences in pain perception or reporting behavior, as female sex has been independently identified as a risk factor for painful—as opposed to painless—DPN (15).

Among the antidiabetic treatment regimens and neurological conditions analyzed, insulin therapy was associated with the highest risk of PDPN. This finding is consistent with previous studies identifying insulin use as a risk factor for DPN. Experimental studies have further suggested that insulin administration may induce significant intraneural hypoxic effects, which could contribute to nerve dysfunction (51).

In summary, this study develops a practical tool for predicting PDPN risk in community-based T2DM patients, identifying several clinically relevant and biologically plausible predictors. The logistic regression model, operationalized as a web-based dynamic nomogram, is recommended as a first-line pre-screening tool for implementation in primary care settings. Its use can facilitate early identification of high-risk individuals, guide targeted specialist referrals, and optimize resource allocation within community health systems. Future work should focus on prospective validation and the integration of this nomogram into Chinese primary care workflows and electronic health records. In addition, longitudinal studies are needed to clarify the causal pathways of the identified predictors.

### Limitations

Despite its strengths, this study has several limitations. First, the retrospective design may introduce information bias and precludes causal inference. Prospective studies are needed to validate predictive associations over time. Second, although the study included multiple centers, all participants were from Beijing, which may limit the generalizability of our findings to other regions or ethnic groups. Moreover, minor differences in gender composition or sample distribution across populations may further influence model generalizability. External validation in independent cohorts across diverse populations is urgently needed to confirm the model’s robustness and applicability. Third, the relatively low prevalence of PDPN (4.08%) may impact model performance, particularly positive predictive value, despite the use of imbalance-handling techniques. Fourth, PDPN diagnosis in this study was based on mTCNS and DN4 scores. Although these tools are validated and widely used in epidemiological research, they are inherently subjective and prone to inter-observer variability, which may introduce misclassification bias. The absence of objective diagnostic modalities such as nerve conduction studies (NCS), regarded as the gold standard, may have led to either underestimation or overestimation of PDPN prevalence. Future investigations incorporating NCS and other objective biomarkers are warranted to enhance diagnostic precision and strengthen external validity. Finally, the dataset lacked detailed information on lifestyle factors, comorbidity duration, medication specifics, and genetic predisposition, which may further improve risk prediction in future models.

## Conclusion

In conclusion, this study developed and validated a multicenter clinical prediction model for PDPN in T2DM patients based on data from 13 community hospitals in Beijing. The RF and LR models, incorporating ten easily obtainable clinical and demographic variables, demonstrated strong predictive performance and good calibration. Key predictors, notably HbA1c, musculoskeletal disorders, DBP, BMI, and hyperlipidemia underscore important clinical considerations. Importantly, this model provides a practical and interpretable tool for early risk stratification and targeted screening of PDPN in primary care and community settings, where specialized neurological assessments are often limited. By integrating this prediction model into routine diabetes management, such as electronic health record systems or web-based nomograms, clinicians can proactively identify high-risk individuals, optimize referral pathways, and support preventive interventions. Such integration may enhance clinical decision-making, promote personalized management, and ultimately reduce the burden of diabetic neuropathy.

## Data Availability

The raw data supporting the conclusions of this article will be made available by the authors, without undue reservation.
