# Supplementary material for: Development and validation of a risk prediction model for painful diabetic peripheral neuropathy in type 2 diabetes mellitus: a multicenter retrospective study
Source: Front Endocrinol (Lausanne). 2025 Nov 27;16:1651493. doi: 10.3389/fendo.2025.1651493 (PMC12696710; doi:10.3389/fendo.2025.1651493)
Supplement: Supplementary file 1 [file DataSheet1.pdf]

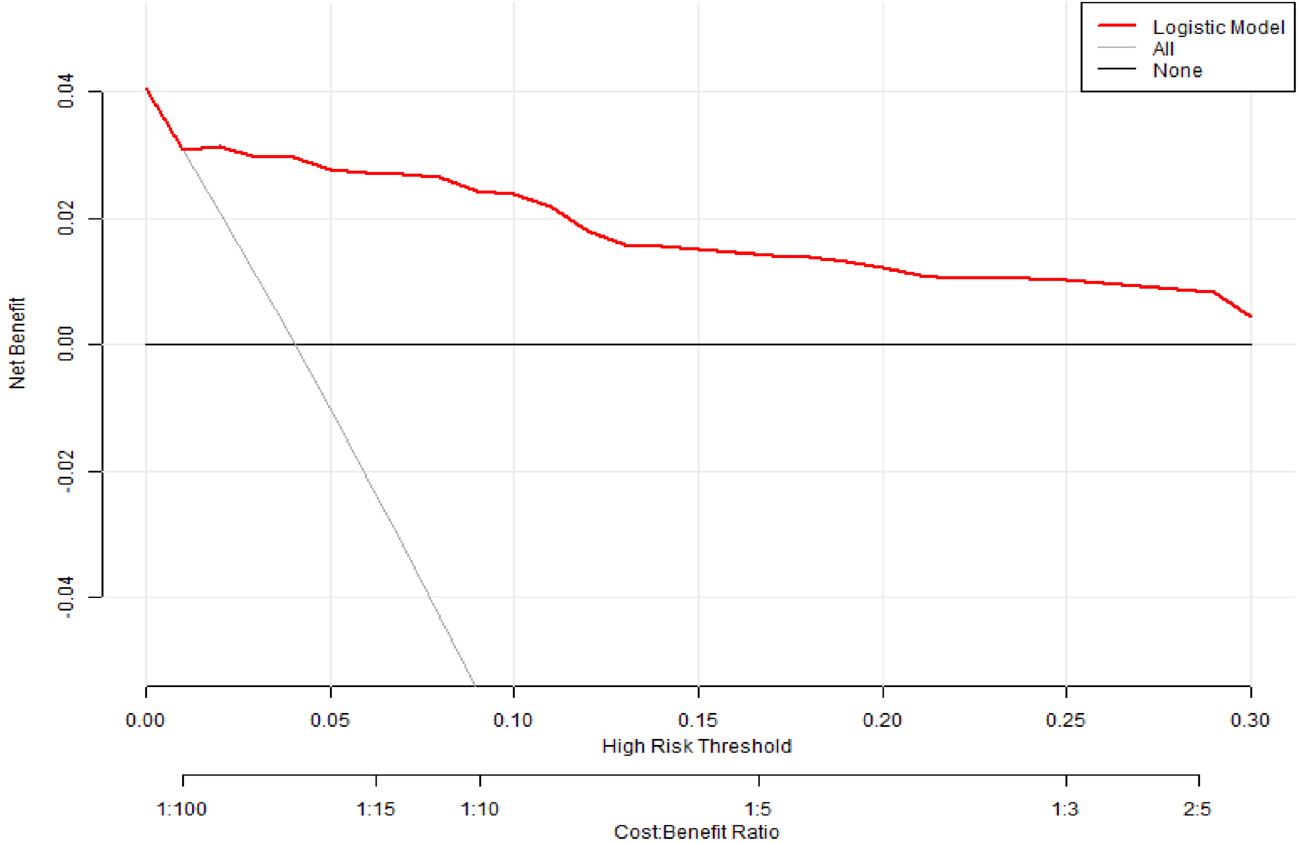

Supplementary figure 1. Decision curve analysis (DCA) for the logistic regression (LR) model.The red line shows the net clinical benefit of the LR model across threshold probabilities. The black line (“ None ”) assumes no patients are treated, and the gray line (“ All ”) assumes all are treated. The LR model yields greater net benefit within clinically relevant thresholds, supporting its utility for risk stratification.
