# Supplementary material for: Development and validation of a risk prediction model for painful diabetic peripheral neuropathy in type 2 diabetes mellitus: a multicenter retrospective study
Source: Front Endocrinol (Lausanne). 2025 Nov 27;16:1651493. doi: 10.3389/fendo.2025.1651493 (PMC12696710; doi:10.3389/fendo.2025.1651493)
Supplement: Supplementary file 2 [file DataSheet2.pdf]

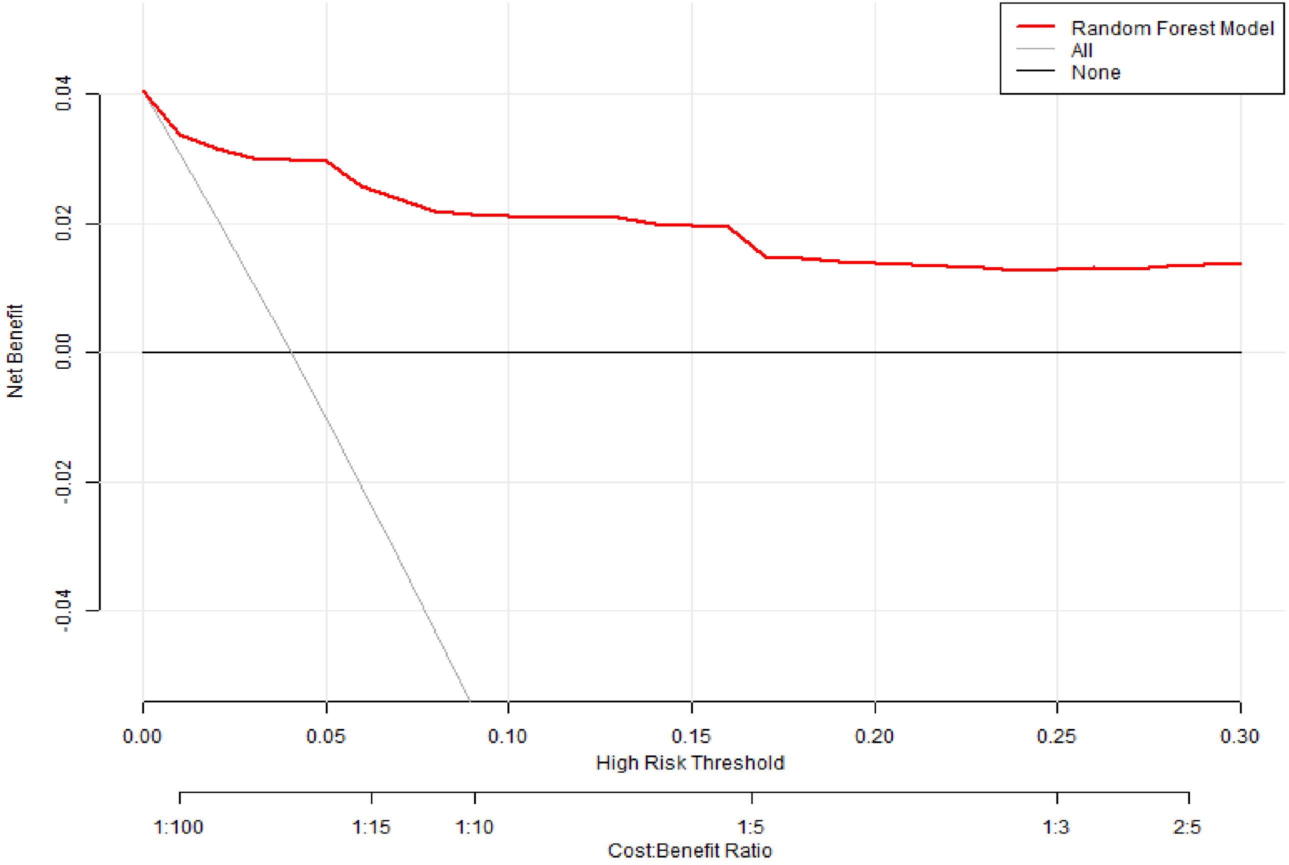

Supplementary figure 2. Decision curve analysis (DCA) for the random forest (RF) model. The RF model (red line) provides greater net benefit than the “treat-none” (black) and “treat-all” (gray) strategies within the threshold range of 0.05 – 0.25, indicating improved clinical decision-making performance.
