# Supplementary material for: Development and validation of a risk prediction model for painful diabetic peripheral neuropathy in type 2 diabetes mellitus: a multicenter retrospective study
Source: Front Endocrinol (Lausanne). 2025 Nov 27;16:1651493. doi: 10.3389/fendo.2025.1651493 (PMC12696710; doi:10.3389/fendo.2025.1651493)
Supplement: Supplementary file 3 [file Table1.docx]

| Supplementary Table 1. Baseline characteristics of participants with T2DM. | | | | | |
| --- | --- | --- | --- | --- | --- |
| Variables | Total (n = 1984) | Test set (n = 594) | Development set (n = 1390) | Statistic | *P* |
|  |  |  |  |  |  |
| Age, M (Q₁, Q₃) | 64.00 (57.00, 70.00) | 64.00 (57.00, 70.00) | 65.00 (57.00, 70.00) | Z=-0.53 | 0.593 |
| Sex, n(%) |  |  |  | χ²=1.92 | 0.166 |
| male | 959 (48.34) | 273 (45.96) | 686 (49.35) |  |  |
| female | 1025 (51.66) | 321 (54.04) | 704 (50.65) |  |  |
| BMI,  M (Q₁, Q₃) | 25.39 (23.44, 27.68) | 25.39 (23.34, 27.72) | 25.39 (23.44, 27.68) | Z=-0.04 | 0.967 |
| Education, n(%) |  |  |  | χ²=6.40 | 0.171 |
| junior high school or below | 947 (47.73) | 279 (46.97) | 668 (48.06) |  |  |
| high school/vocational/technical school | 570 (28.73) | 166 (27.95) | 404 (29.06) |  |  |
| associate degree | 242 (12.20) | 80 (13.47) | 162 (11.65) |  |  |
| bachelor's degree | 204 (10.28) | 67 (11.28) | 137 (9.86) |  |  |
| postgraduate or above | 21 (1.06) | 2 (0.34) | 19 (1.37) |  |  |
| Marital Status, n(%) |  |  |  | χ²=2.43 | 0.489 |
| unmarried | 21 (1.06) | 8 (1.35) | 13 (0.94) |  |  |
| married | 1881 (94.81) | 566 (95.29) | 1315 (94.60) |  |  |
| widowed | 70 (3.53) | 16 (2.69) | 54 (3.88) |  |  |
| divorced | 12 (0.60) | 4 (0.67) | 8 (0.58) |  |  |
| Physical activity, n(%) |  |  |  | χ²=0.59 | 0.745 |
| low-intensity | 1706 (85.99) | 506 (85.19) | 1200 (86.33) |  |  |
| moderate-intensity | 270 (13.61) | 85 (14.31) | 185 (13.31) |  |  |
| high-intensity | 8 (0.40) | 3 (0.51) | 5 (0.36) |  |  |
| Smoking History, n(%) |  |  |  | χ²=2.54 | 0.111 |
| yes | 401 (20.21) | 107 (18.01) | 294 (21.15) |  |  |
| no | 1583 (79.79) | 487 (81.99) | 1096 (78.85) |  |  |
| Alcohol Consumption History, n(%) |  |  |  | χ²=0.35 | 0.553 |
| yes | 485 (24.45) | 140 (23.57) | 345 (24.82) |  |  |
| no | 1499 (75.55) | 454 (76.43) | 1045 (75.18) |  |  |
| Duration, M (Q₁, Q₃) | 98.00 (45.00, 163.00) | 93.00 (40.25, 158.00) | 98.00 (48.00, 164.00) | Z=-1.78 | 0.075 |
| HbA1c, M (Q₁, Q₃) | 6.50 (6.00, 7.00) | 6.50 (6.00, 7.00) | 6.50 (6.00, 7.00) | Z=-1.02 | 0.309 |
| FPG, n(%) |  |  |  | χ²=6.31 | 0.098 |
| <6 mmol/L | 268 (13.51) | 87 (14.65) | 181 (13.02) |  |  |
| 6–7 mmol/L, , | 942 (47.48) | 288 (48.48) | 654 (47.05) |  |  |
| 7–8 mmol/L | 521 (26.26) | 135 (22.73) | 386 (27.77) |  |  |
| >8 mmol/L | 253 (12.75) | 84 (14.14) | 169 (12.16) |  |  |
| PPG, n(%) |  |  |  | χ²=1.24 | 0.744 |
| <8 mmol/L | 433 (21.82) | 139 (23.40) | 294 (21.15) |  |  |
| 8–10 mmol/L | 905 (45.61) | 265 (44.61) | 640 (46.04) |  |  |
| 10–11.1 mmol/L | 397 (20.01) | 117 (19.70) | 280 (20.14) |  |  |
| >11.1 mmol/L | 249 (12.55) | 73 (12.29) | 176 (12.66) |  |  |
| Blood glucose self-monitoring, n(%) |  |  |  | χ²=0.00 | 0.993 |
| yes | 1446 (72.88) | 433 (72.90) | 1013 (72.88) |  |  |
| no | 538 (27.12) | 161 (27.10) | 377 (27.12) |  |  |
| Antidiabetic treatment regimen, n(%) |  |  |  | χ²=0.91 | 0.824 |
| oral hypoglycemic agents | 1491 (75.15) | 452 (76.09) | 1039 (74.75) |  |  |
| insulin | 91 (4.59) | 29 (4.88) | 62 (4.46) |  |  |
| both | 259 (13.05) | 73 (12.29) | 186 (13.38) |  |  |
| none | 143 (7.21) | 40 (6.73) | 103 (7.41) |  |  |
| SBP, M (Q₁, Q₃) | 130.00 (123.00, 134.00) | 130.00 (122.00, 134.00) | 130.00 (123.00, 134.00) | Z=-0.25 | 0.806 |
| DBP, M (Q₁, Q₃) | 80.00 (74.00, 81.25) | 80.00 (75.00, 81.00) | 80.00 (74.00, 81.75) | Z=-0.90 | 0.368 |
| Metabolic Syndrome, n(%) |  |  |  | χ²=0.20 | 0.656 |
| yes | 1144 (57.66) | 347 (58.42) | 797 (57.34) |  |  |
| no | 840 (42.34) | 247 (41.58) | 593 (42.66) |  |  |
| Hypertension, n(%) |  |  |  | χ²=0.37 | 0.542 |
| yes | 1485 (74.85) | 450 (75.76) | 1035 (74.46) |  |  |
| no | 499 (25.15) | 144 (24.24) | 355 (25.54) |  |  |
| Cardiovascular Disease, n(%) |  |  |  | χ²=1.15 | 0.283 |
| yes | 555 (27.97) | 176 (29.63) | 379 (27.27) |  |  |
| no | 1429 (72.03) | 418 (70.37) | 1011 (72.73) |  |  |
| Musculoskeletal Disorders, n(%) |  |  |  | χ²=0.29 | 0.590 |
| yes | 115 (5.80) | 37 (6.23) | 78 (5.61) |  |  |
| no | 1869 (94.20) | 557 (93.77) | 1312 (94.39) |  |  |
| Neurological Disorders, n(%) |  |  |  | χ²=0.23 | 0.634 |
| yes | 58 (2.92) | 19 (3.20) | 39 (2.81) |  |  |
| no | 1926 (97.08) | 575 (96.80) | 1351 (97.19) |  |  |
| Hyperlipidemia, n(%) |  |  |  | χ²=0.00 | 0.990 |
| yes | 701 (35.33) | 210 (35.35) | 491 (35.32) |  |  |
| no | 1283 (64.67) | 384 (64.65) | 899 (64.68) |  |  |
| Rheumatology Conditions, n(%) |  |  |  | χ²=5.48 | **0.019** |
| yes | 23 (1.16) | 12 (2.02) | 11 (0.79) |  |  |
| no | 1961 (98.84) | 582 (97.98) | 1379 (99.21) |  |  |
| PDPN, n(%) |  |  |  | χ²=0.00 | 0.950 |
| yes | 1903 (95.92) | 570 (95.96) | 1333 (95.90) |  |  |
| no | 81 (4.08) | 24 (4.04) | 57 (4.10) |  |  |
| Abbreviations: Z: Mann-Whitney test, χ²: Chi-square test, M: Median, Q₁: 1st Quartile, Q₃: 3rd Quartile, BMI: Body Mass Index, HbA1c: Hemoglobin A1c, FPG: Fasting Plasma Glucose, PPG: Postprandial Plasma Glucose, SBP: Systolic Blood Pressure, DBP: Diastolic Blood Pressure, PDPN: Painful Diabetic Peripheral Neuropathy | | | | | |
