# Supplementary material for: Development and validation of a risk prediction model for painful diabetic peripheral neuropathy in type 2 diabetes mellitus: a multicenter retrospective study
Source: Front Endocrinol (Lausanne). 2025 Nov 27;16:1651493. doi: 10.3389/fendo.2025.1651493 (PMC12696710; doi:10.3389/fendo.2025.1651493)
Supplement: Supplementary file 4 [file Table2.docx]

**Supplementary table 2: Comparison of predictive performance between the original and re-randomized models.**

| Models | ROC.AUC(95% CI) | PR.AUC(95% CI) | Brier Score | G-Mean | Sensitivity | Specificity | PPV (Precision) | NPV | PPV/NPV | F1 Score | recall | Balanced Accuracy | MCC |
| --- | --- | --- | --- | --- | --- | --- | --- | --- | --- | --- | --- | --- | --- |
| LR_original | 0.894 (0.814–0.964) | 0.470 (0.258–0.665) | 0.038 (0.027–0.051) | 0.807 (0.577–0.932) | 0.688 (0.333–0.913) | 0.965 (0.936–0.998) | 0.514 (0.306-0.923) | 0.987 (0.973-0.996) | 0.521 | 0.549 (0.414–0.686) | 0.695 | 0.826 (0.667–0.932) | 0.541 |
| LR_re-randomized | 0.922 (0.878-0.959) | 0.363 (0.183-0.564) | 0.037 (0.027-0.048) | 0.756 (0.592-0.881) | 0.598 (0.357-0.807) | 0.967 (0.934-0.991) | 0.444 (0.270-0.679) | 0.983 (0.972-0.993) | 0.45 | 0.499 (0.345-0.640) | 0.6 | 0.782 (0.670-0.885) | 0.48 |
| RF_original | 0.913 (0.834-0.974) | 0.488 (0.276-0.694) | 0.029 (0.021-0.039) | 0.744 (0.591-0.889) | 0.571 (0.353-0.833) | 0.983 (0.936-0.998) | 0.652 (0.333-0.941) | 0.982 (0.970-0.991) | 0.664 | 0.582 (0.419-0.739) | 0.575 | 0.777 (0.672-0.892) | 0.56 |
| RF_re-randomized | 0.926 (0.879-0.964) | 0.421 (0.218-0.627) | 0.033 (0.024-0.043) | 0.735 (0.576-0.863) | 0.559 (0.333-0.762) | 0.977 (0.950-0.995) | 0.525 (0.300-0.750) | 0.982 (0.970-0.991) | 0.53 | 0.531 (0.355-0.688) | 0.5602 | 0.768 (0.660-0.870) | 0.513 |
| **Abbreviations:** LR: multivariable Logistic Regression, RF: Random Forest, AUC-ROC: Area Under the Receiver Operating Characteristic Curve, CI: Confidence Interval, PR-AUC: Area Under the Precision-Recall Curve, PPV: Positive Predictive Value, NPV: Negative Predictive Value, MCC: Matthews Correlation Coefficient | | | | | | | | | | | | | |
|  | | | | | | | | | | | | | |
